# A cross sectional survey on the effect of COVID-19 related restrictions on undergraduate and postgraduate medical education in Qatar

**DOI:** 10.1186/s12909-022-03268-z

**Published:** 2022-03-29

**Authors:** M Thomas, S Suliman, M Allen, M Hameed, A Ghaffar, MM Emara, H Fatima, S George, R Singh, H Ghazouaini, AL Alkhal

**Affiliations:** 1 Department of Chest, Hamad General Hospital, Doha, Qatar; 2 Department of Clinical Medicine, Weill Cornell Medical College, Ar-Rayyan, Qatar; 3 Department of Medicine, Hamad General Hospital, Doha, Qatar; 4 Qatar University, College of Medicine, Doha, Qatar; 5 Medical Education Department, Hamad General Hospital, Doha, Qatar; 6 Medical Research Centre, Hamad General Hospital, Doha, Qatar

**Keywords:** COVID-19, Medical education, Survey

## Abstract

**Background:**

COVID-19 pandemic has affected all dimensions of day to day life across the world and medical education was no exception. With this study, we aimed to understand the effect of nationwide restrictions on medical education in Qatar, the models of education adopted during this period and perceptions of participants to the same.

**Methods:**

We conducted a cross-sectional study utilizing an online questionnaire distributed via convenience sampling between April–October 2020. Study participants were faculty and trainees in governmental undergraduate and postgraduate medical education institutes. Two sets of questionnaires were designed for each group. They were asked a series of questions to assess pre- and post-COVID pandemic educational practices, their preferred teaching methods, and their familiarity with electronic teaching platforms. Faculty respondents were asked about their perceived barriers to delivery of medical education during the pandemic and their agreement on a 5-point Likert scale on specific elements. Trainees were asked a series of multiple-choice questions to characterize their pre- and post-COVID pandemic educational experiences. Both groups were asked open-ended questions to provide qualitative insights into their answers. Data were analysed using STATA software version 12.0.

**Results:**

Majority of trainees (58.5%) responded that the pandemic has adversely affected medical education at both the undergraduate and postgraduate levels. Trainees (58.5%) and faculty (35.7%) reported an increased reliance on e-learning. Trainees preferred face-to-face education, while faculty preferred a combination of models of education delivery (33.5% versus 37.1%, *p* = 0.38). Although 52.5% of the faculty had no previous experience of delivering education through e-learning modalities, 58.9% however felt confident in using e-learning software.

**Conclusions:**

Faculty and trainees agree that the COVID-19 pandemic has had a significant impact on the provision of medical education and training in Qatar, with an increased dependence on e-learning. As trainee’s prefer face-to-face models of education, we may have to consider restructuring of medical curricula in order to ensure that optimum learning is achieved via e-learning, while at the same time enhancing our use, knowledge and understanding of the e -learning methods. Further research is warranted to assess if these changes have influenced objective educational outcomes like graduation rates or board scores.

**Supplementary Information:**

The online version contains supplementary material available at 10.1186/s12909-022-03268-z.

## Introduction

The year 2020 will be synonymous with the acronym COVID-19. The SARS-CoV-2 virus spread rapidly across continents, resulting in the COVID-19 pandemic [1]. As the virus demonstrated quick and efficient human-to-human transmission even from those who were asymptomatic, WHO recommended social distancing, defined as maintaining at least 6 feet of distance between people and avoiding large crowds and public places [2, 3]. This important measure has led to businesses, economies and institutions adapting to new models of working and sustainability; medical education was no exception.

Nationwide lockdown restrictions were implemented in Qatar on March 9, 2020 resulting in the closure of schools and universities [4]. In the state of Qatar, two institutions are providing undergraduate medical education, Qatar University—College of medicine (QU CMED) and Weill Cornell Medical College, Qatar (WCMQ). Post Graduate Medical Education is provided by the teaching hospitals under the government run Hamad Medical Corporation (HMC) through residency and fellowship training programs (ACGME-I accredited). HMC implemented stringent infection control measures including restricted entry to visitors, trainees, and outpatients, with the introduction of telemedicine for many outpatient encounters. Clinical and non-clinical staff were mobilised to three new COVID-19 designated hospitals to deal with the unprecedented burden of the pandemic [5]. For much of this extraordinary effort, HMC stopped or restricted most routine educational activities.

Hands on learning has always been at the heart of medical education but the pandemic's rapid evolution meant critical decisions about medical education had to be made without significant input from either students/trainees or faculty. Traditional methods of teaching and training had to be discontinued or replaced with e-learning or online learning [6, 7]. We conducted a cross-sectional study using an online survey to better understand the impact of these abrupt changes on the traditional methods of medical education, their continuity as a result of healthcare workforce deployment to COVID hospitals, and the gaps it has created in learning. We also looked at trainees and faculty's perceptions of the new medical education delivery models that were implemented which can be used to model and revise conventional methods to address the gaps based on their needs and goals. The survey was administered to two cohorts, one being the undergraduate medical trainees and faculty at QU CMED, and the second being the postgraduate trainees and faculty of ACGME-I accredited programs at HMC.

## Materials and methods

This was a cross-sectional study conducted utilising an online survey questionnaire between April and October 2020.

### Questionnaire design and distribution

Questionnaires were designed to gather precise information and insight into the effects of the current ongoing global COVID-19 pandemic on medical education. Two sets of questions were framed for the target participants who were categorized into two pre-defined groups, one consisting of trainees and the other consisting of faculty. The initial trainee and faculty questionnaires consisted of 24 questions, which were reduced to 18 questions following an expert panel discussion. This was done by combining some of the questions and removing others of less relevance. The format consisted of binary, multiple-choice and Likert scale type questions. However, for some questions a descriptive answer could be provided by choosing the option “Other: Please specify”.

Both trainee and faculty questionnaires were submitted for content validation to five experts in the field of medical education who reviewed for content validity and construct validation was performed using Principal Component Analysis (PCA) [8]. Content validity indices were computed based on the expert's rating of the item’s relevance towards the objectives of the study. Each item was graded as, not relevant, somewhat relevant, quite relevant, or highly relevant. The calculated Item-content validity index (I-CVI) across all items was 0.95 for both faculty and trainee surveys. The Scale-level content validity index based on the universal agreement method (S-CVI/UA) for faculty and trainee surveys across all items was 0.8 and 0.7 respectively. Further changes were made to the questionnaire based on the expert recommendations to reach I-CVI of 1.0 and S-CVI/UA ≥ 0.8. Construct validity by exploratory analysis showed that the model explained 90.5% variance for the latent variable educational activity with 8 components in faculty survey and 85.8% variance for 6 components in trainee survey demonstrated in scree plot S1 Figure. The final version consisted of 13 questions for the faculty and 11 questions for the trainees. The final survey explored three themes: 1. General demographics 2. Status of medical training, and 3. Models of education: Perceptions and preferences with focus on e-learning. The responses were collected and categorised after thematic analysis.

To assess test—retest variability, the questionnaire was piloted among fifteen participants for each survey. These participants included trainee and faculty fulfilling inclusion exclusion criteria selected by convenience sampling. This was done twice fifteen days apart to evaluate if the answers given, matched their earlier responses. The Cronbach’s alpha for all items was 0.98 for faculty survey and 0.981 for trainee survey indicating a good level of internal consistency. The results of the pilot validation were not included in the final analysis.

The survey was created using Qualtrix, an online survey software. A convenience sampling technique was employed to obtain response from participants. The validated survey was distributed via email to all participants by the secretary in-charge for QU-CMED and HMC Residency programs. Social media platforms were utilised for marketing only. The survey was accessible via an anonymous link for a 2-week period from September 10,2020. This was followed by 3 reminders to the participant’s email. At the end of the 2-week period the survey was automatically disabled.

### Participants

The inclusion criteria mandated participants were either medical trainees/students or teaching faculty from the institutions below:

A) Medical trainees falling under any one of the following categories.

1—Qatar University College of Medicine medical students.

2-ACGME-I accredited Residency programs under Medical Education, HMC.

3- ACGME-I accredited Fellowship programs under Medical Education, HMC.

B. Teaching faculty under the following.

1- Qatar University College of Medicine.

2- Faculty in ACGME-I accredited Residency programs under Medical Education, HMC.

3- Faculty in ACGME-I accredited Fellowship programs under Medical Education, HMC.

Exclusion criteria included learners in clinical attachment and clerkship rotations.

### Ethical considerations

Participation in the research was voluntary. All data collected was anonymous. Consent and single submission of the survey by each participant was through 2 initial mandatory questions. The study was ethically approved by the Institutional Review Boards of Hamad Medical Corporation (#MRC-01–20-1068) and Qatar University.

### Data analysis

#### Quantitative analysis

Data recorded in Qualtrix survey software was exported to Microsoft Excel (Excel Version 2008). Descriptive statistics were used to summarize and determine the sample characteristics and distribution of various considered parameters. Frequencies and proportions were used for categorical variables. Associations between groups were evaluated with Chi [2] test or Fisher exact test for categorical variables. *P*-value < 0.05 (two tailed) was considered statistically significant. STATA software version 12.0 (Stata corp, College Station, TX, USA) was used for exploratory data analysis in the form of Principal Component Analysis (PCA) and descriptive statistics and Sigma Plot software was used to create graphs. At the time of statistical analysis, missing data were handled primarily by complete case analysis.

#### Qualitative analysis

We performed a thematic analysis of the participants’ comments in the open-ended questions in the survey manually on an excel sheet. SS reviewed the comments of participant surveys and coded the first 50 comments and drafted the initial coding scheme. MM and SS reviewed the coding scheme and adjusted it to generate the themes. Thereafter SS completed the coding process and generated a coding scheme that included categories and codes that emerged. Together the two authors conducted constant comparison of emerging characteristics and properties of the category and among different perceptions and readings of the data [9, 10].

## Results

### Trainee survey

#### Demographics

The trainee survey was distributed to 324 medical students and 796 post graduate trainees. The response rate was 156 (48%) from medical students and 193 (24%) from post graduate trainees (Table 1).

**Table 1:** Demographics of Faculty and Trainee for Survey

Institution	Respondents N (%) *Institutional Count (%)*
**Faculty Survey**	
Hamad Medical Corporation	191(91.4) *(31.8)*
Qatar University (Medicine)	18 (8.6) *(45)*
**Trainee Survey**	
Medical student	156(44.8) *(48)*
Post graduate trainees (total)	192 (55.1) *(24)*
Residency	143 (41.1)
Fellowship	49 (14.0)
Surgical speciality	48 (25.0)
Medical speciality	118 (62.0)
Other specialty	26 (13.5)

#### Barriers to education and adaptation to COVID 19

**Fig. 1:**
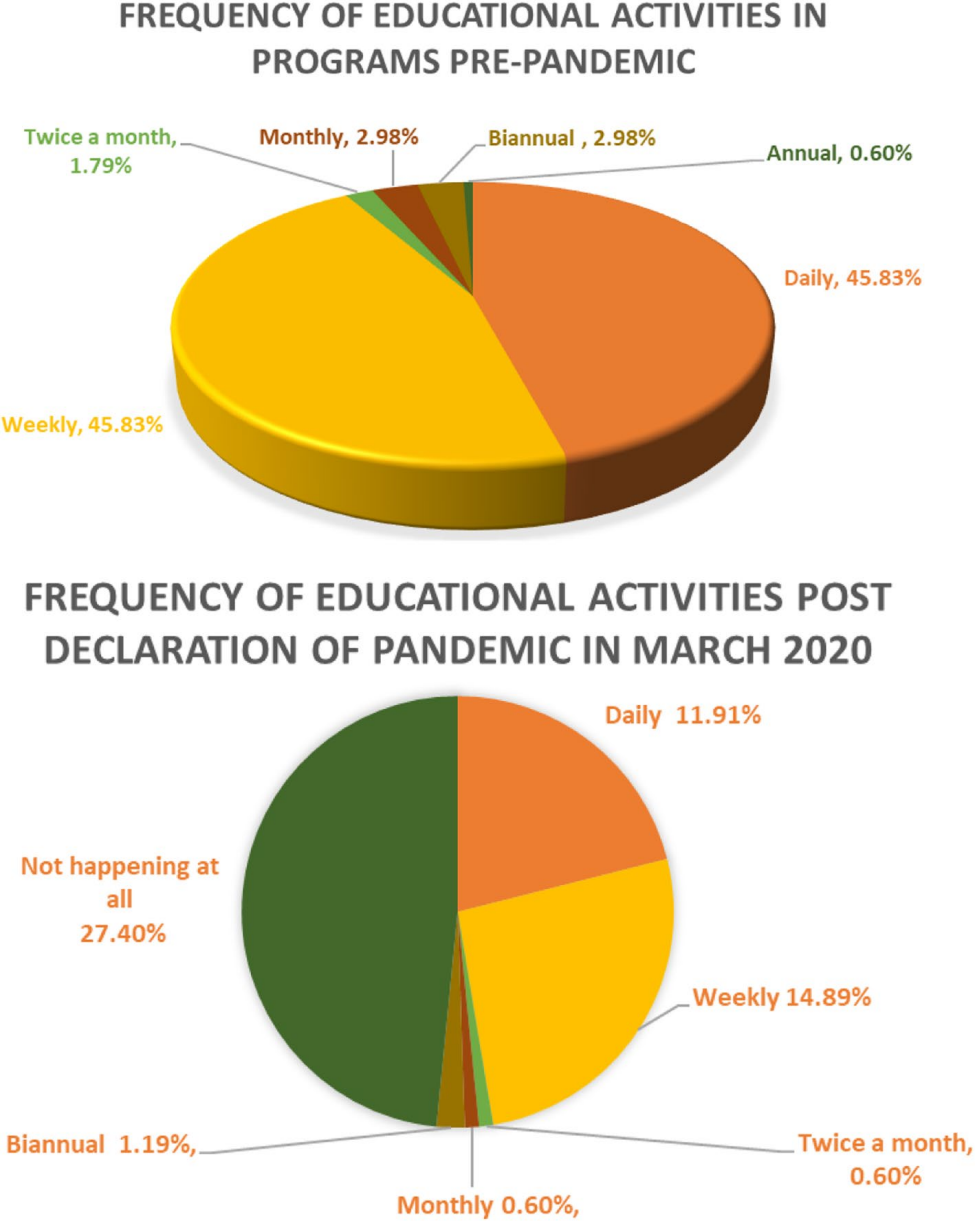
Frequency of educational activities before and after COVID

A majority of the trainees (58.5%) responded that the pandemic has adversely affected their training, with no significant difference between medical students and post graduate trainees of surgical or medical specialities (*p* = 0.208). Their specific reasons are categorized in Table 2. Of the trainees, 49% reported a suspension of face-to-face educational activities due to the pandemic, while 7.4% reported not being able to attend due to serving at the ‘front line’. Only 12.6% of trainees reported a complete cessation of all forms of educational activities. There was a significant drop in the frequency of all educational activities depicted in Fig. 1, at both undergraduate and post graduate levels during the survey period (*p* < 0.001).

**Table 2:** Impact of the current COVID-19 pandemic on trainees

Theme	Details	Exemplar Quotations
Scope of practice	This theme describes the change in the scope of service of the postgraduate trainees as majority had been deployed to COVID-19 facilities	*“Working in COVID-19 dedicated facilities for months, working with similar patients without diversity in cases.” (R)* *“I was deployed to COVID-19 facility and missed 3 blocks of my rotation.” (R)*
Case Load profile	This theme describes the trainees’ perceptions of the impact of the pandemic on the trainees’ exposures to specialty specific cases	*“Less exposure to cases either Emergency or Elective as it was cancelled due to COVID-19, no face-to-face educational activities” (S)* *“Less number of elective surgical cases and theatre workload.” (R)*
Education (Theoretical)	This theme describes the trainees’ perceptions of the impact of the pandemic on the trainees’ theoretical education such as face-to-face activities and the difficulty to engage with online activities	*“Many courses have postponed, activities cancelled.” (S)* *No morning report or direct supervision by consultants.” (R)*
Education (Practical)	This theme describes the trainees’ perceptions of the impact of the pandemic on practical education such as bedside teaching and clerkship rotations	*“no education rounds, less procedures no courses no educational days….” (R)* *“Clinical skills classes have been cancelled.” (S)*
Impact on Learning Spaces	This theme describes the trainees’ perceptions of the impact of the pandemic on the availability of learning spaces such as libraries and study lounges	*I can’t visit campus all lessons are online even the practical ones which makes it hard especially for clinical practice sessions and lab sessions.” (S)*
Impact on communication and interaction	This theme describes the trainees’ perceptions of the impact of the pandemic on trainees’ interaction with patients, colleagues, and supervisors	*“We lost our connection with our program as we all worked in separate places.” (R)* *“Decreased my interactions with patients and other health care professionals so I feel my clinical skills were not as sharp as before.” (R)*
Workload	This theme describes the trainees’ perceptions of the impact of the pandemic on pandemic-related workload	*“Too much workload and busy calls.” (R)* *“Working hours have increased with less time allocated for teaching.” (R)*
Travel and infection Control Restrictions	This theme describes the trainees’ perceptions of the impact of the pandemic on travel and infection control restrictions	*“No leave, more stress.” (R)* *“Stress and fear of infection affected the Interest to be in contact with the patients.” (R)*

#### Results of the qualitative analysis

Trainees’ comments on the impact of the COVID-19 pandemic on their training revealed eight themes summarized in Table 2. The change in the scope of service with postgraduate trainees being deployed to COVID facilities resulted in many missing planned rotations. At the peak of the pandemic medical students were not allowed to enter hospitals resulting in shortening or cancellation of clerkship rotations. One dramatic effect was the fast and significant shift in general of undergraduate educational activities to online. However, almost all postgraduate educational programs stopped completely for at least the initial few months of the pandemic, with a detrimental effect on practical bedside and procedural skills teaching. Because of the fear of COVID-19 infection, trainees also had less exposure to patients and less interaction with colleagues and supervisors, thereby decreasing experiential learning. In the supporting quotes, residents are identified with (*R*) and students with (*S*).

#### Models of education and perceptions

Since the start of the pandemic educational activities were delivered predominantly online with 57% of the trainees receiving e-learning. Of these, the majority received live online lectures 65.6%, followed by broadcast of recorded lectures 20.1% and sharing of learning material links 23.5%. Given the experience of online teaching during the COVID-19 pandemic, only 7.1% preferred e-learning alone, whereas 33.5% of trainees still prefer face to face education, followed by 26.1% preferring a combination of face to face learning combined with either synchronous or asynchronous e-learning or self-directed learning strategies. The reasons of trainee’s preference for face to face learning are summarized in S1 Table, where interaction with mentors, peers and activity engagement stood out as the most important reasons. These results were confirmed by the trainees ranking their perceptions of e-learning on a Likert scale ranging from Strongly agree to Strongly disagree (*n* = 186). E-learning was also perceived to be less effective than face to face learning by 53.8% trainees, whereas 33.3% agreed to strongly agreed that e-learning is as effective as traditional methods of delivery of education. Moreover, 56.9 and 32.2% trainees showed a level of agreement ranging from agree to strongly agree in stating that e-learning enhances their knowledge and improves their communication skills, respectively. On the effectiveness of e-learning in enhancing knowledge, postgraduate trainees had a significantly higher level of agreement compared to trainees (74.4% vs 39%, *p* < 0.00001). However, 58.6% agreed that e-learning is effective in integrating different multimedia forms of delivering quality education.

#### Faculty survey

The faculty survey was distributed to 40 members of QU faculty and 600 HMC faculty staff with a response rate of 45 and 31.8% respectively (Table 1).

#### Demographics

As HMC postgraduate faculty may also teach at QU there is an overlap in faculty affiliations, however the majority of respondents were faculty at HMC (Table 1). The postgraduate trainee target group of learners taught by the faculty included: Residents 139 (68.8%), Fellows 122 (60.4%); Medical students 79(39.1) and other faculty 99 (49%).

#### Challenges in providing medical education during COVID 19

**Fig. 2:**
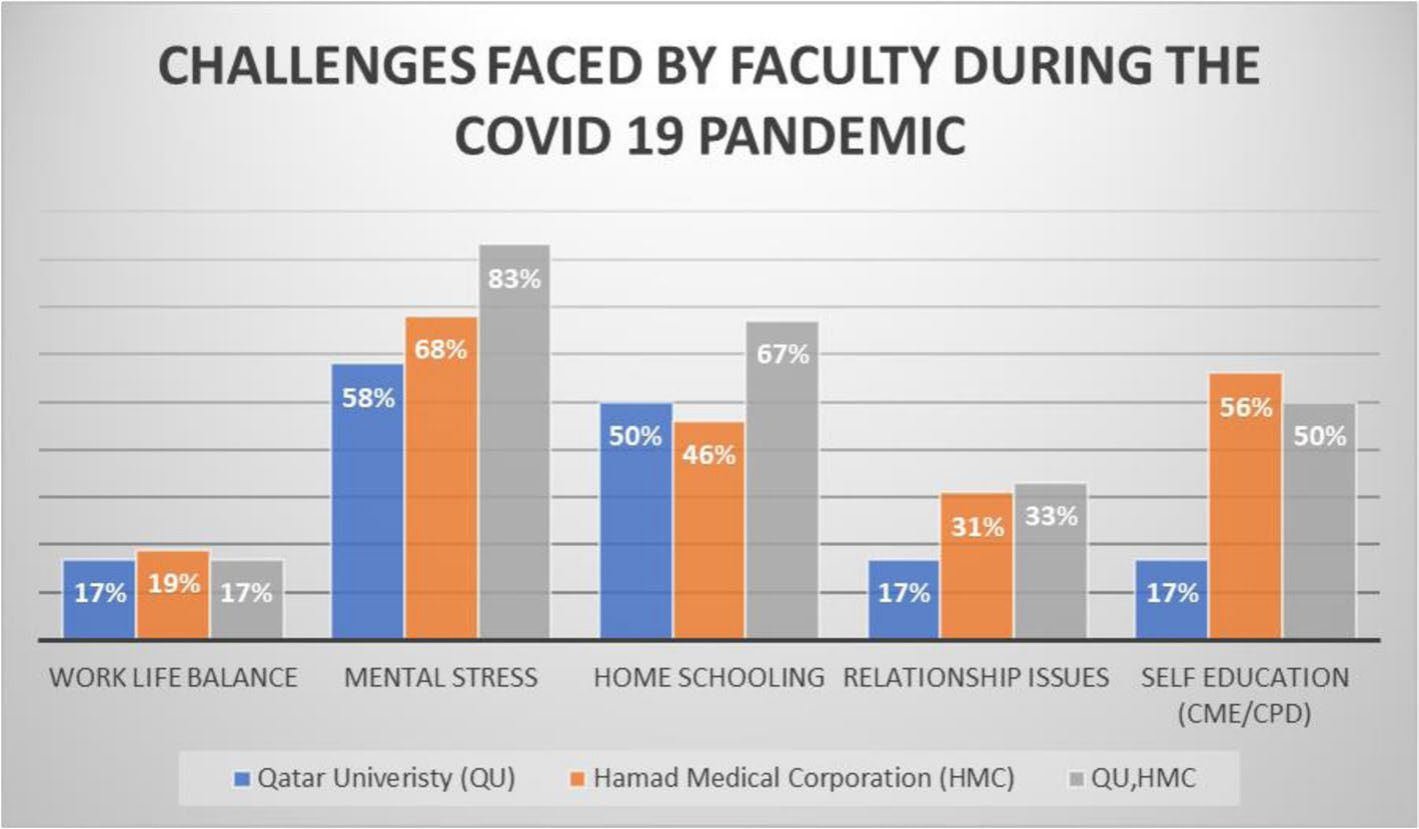
Frequency of educational activities before and after COVID

Similar to trainee’s results, faculty members reported a decreased frequency of educational activities with complete cessation of sessions during the pandemic as reported by 22.8% of the faculty members. Majority of the faculty, 82.7%, agreed to strongly agreed that there were barriers to delivering medical education during the pandemic due to: (1) implementation of social distancing measures (54.9%), (2) unavailability of trainees at the bedside or in a classroom (50.5%), (3) reprioritization of clinical tasks (50%), (4) infection control policies (47%), and (5) lack of time during the pandemic (30.7%). However, the majority, 67%, agreed that service delivery and medical education both are equally important during the pandemic. Importantly, the most common challenges faced by the faculty during the pandemic were mental stress (66.8%), management of work life balance (56.9%), and staying up to date with the continuing professional development requirements (53%) (Fig. 2).

#### Models of education and perceptions

Prior to the pandemic, the most common model of delivery of education was face-to-face activities including didactic lectures, workshops, seminars, journal clubs and bedside teaching while e-learning was utilised in 16.3% activities only. However, since the start of the pandemic, there was an increased reliance on e-learning with 19.3% synchronous lectures, 4% asynchronous activities and 12.4% electronic provision of learning materials. Didactic and bedside teaching reduced to 9.9%. Self-directed learning was encouraged by 17.3% of the faculty. The most effective teaching modality from the faculty’s view was perceived to be a combination of face to face, self-directed and e—learning 37.1%, whereas 34.2% felt that face to face learning alone is the most effective form of educational delivery. The detailed reasons are listed in S2 Table.

**Fig. 3:**
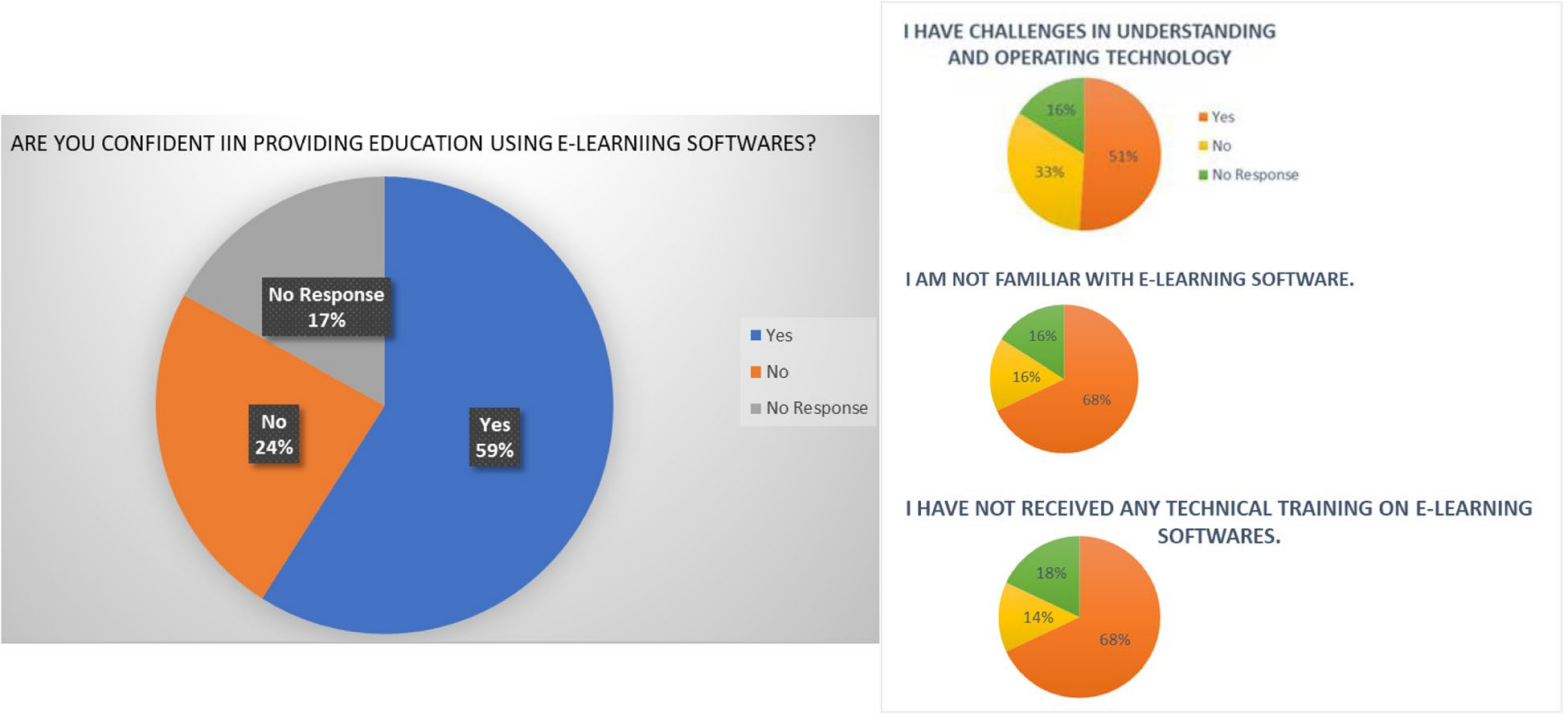
Faculty’s confidence in providing education via e-learning

Although 52.5% of the faculty had no previous experience of delivering education using e-learning modalities, 58.9% however, felt confident in using e-learning software. (Fig. 3) One of the benefits of e-learning reported by the faculty was their ability to provide course material to both learners and their colleagues (72.8%). Other reported benefits included real time presentations to the students 69.3%, developing learner’s understanding of the subject 65.4%, and the ability to create innovative teaching materials 64.9%. E-learning was also felt to be an effective tool to communicate with learners outside the classroom (65.4%), and a way of providing one-to-one attention for learners (46%). Ease of testing learners (49%), tracking their progress (49.5%) and managing individual targets for learners (48%) also showed a high degree of agreement. Some faculty members also highlighted their scepticism or concerns in using e-learning methods which are elaborated in S3 Table.

## Discussion

Our survey highlights that the COVID-19 pandemic has had a significant impact on the continuity of medical education at both undergraduate and postgraduate institutions. Not only the frequency of educational activities but their mode of delivery was affected. This is not surprising considering the nationwide restrictions imposed to reduce the burden of the disease.

Although initially educational activities were temporarily halted completely, the majority of the postgraduate and undergraduate educational institutions adapted very quickly and restarted through virtual or e-learning platforms after a short break. This adaptation to provide continuing education via e-learning has been an international strategy. In the UK, medical colleges also provided virtual learning [11], however, a survey of final year UK medical students reported 77.3% medical elective placements being cancelled with only 1.6% being replaced by formative online modules [12]. Similarly, at a postgraduate level residency training programs in Canada [13], ACGME accredited residency programs in the US [14] and speciality residency programs like neurology [15] started relying on e-learning tools for continuing didactic lectures and providing training in skills and procedures. Our study has also shown the same trend with QU CMED and HMC moving from a predominantly face-to-face delivery of education to online learning albeit after a short break. This is understandable as organisations needed some time to develop and establish remote learning capabilities and protocols.

This paradigm shift in the mode of delivery of medical education through a virtual environment was very swift and hence the long-term realization of its efficacy is crucial to medical education. Both undergraduate and postgraduate medical trainees in our survey considered face-to-face education to be the most effective mode of delivery. Similarly, in a survey of dental students from Indonesia, only 44.2% preferred distance learning to classroom learning [16]. Medical students in the UK also shared similar views of not finding e-learning enjoyable or engaging and a majority found it less effective than face-to- face education [17]. In a survey of Libyan medical students only 38.2% agreed that e-learning can replace traditional models of teaching, although 54% agreed that interactive discussions were possible via e learning [18]. Trainees in our survey also agreed that e-learning encourages communication and interaction while integrating different forms of media. The anonymity that virtual environments provide, may encourage trainees to interact and engage during synchronous e-learning activities, unlike face-to-face learning, where the environment may be intimidating. In our survey post graduate trainees gave a more favourable response to the effectiveness of online learning in enhancing knowledge compared to the medical students (74.4% vs 39%, *p* < 0.00001). This was surprising as postgraduate trainees have predominantly practice-based learning that combines theory, procedures, and clinical skills for a comprehensive training while our early years undergraduate curriculum is mainly focused on the acquisition of content knowledge. Numerous challenges were also faced by the medical fraternity affecting postgraduate medical education during the pandemic. A shortage of personal protective equipment and lack of sufficient testing in some nations contributed to trainee’s reluctance to actively participate in training. Another factor contributing to lesser clinical exposure was the significant drop in the number of patients seeking medical care for conditions other than COVID-19. Telemedicine was adopted as a novel care delivery method across many nations including Qatar for non-urgent medical ailments, which further limited the direct contact and assessment of trainees. Trainees were therefore being taught mostly through e-learning platforms rather than in a clinical environment. This transformation of delivery of skills and training during the COVID-19 pandemic may well play a role in medical training and practice of the future, especially for this generation of trainees. A survey of US medical students has shown that two out of five students feel that changes in medical education during the COVID-19 pandemic will impact their ability to practice medicine [19]. Hence, it becomes key to segregate objectives that can be delivered well online and deliver them during the restrictions, while postponing the objectives that require a physical presence such as clinical examinations and patient contact in the hope of the pandemic easing. Curricula adaptations must ensure that the standard of medical education is not lowered while ensuring that the frontline workforce is being trained to develop the skills and competencies required to serve during the pandemic and well beyond.

Although trainees in our survey did not lean towards a strong agreement on the effectiveness of virtual learning, faculty on the other hand found it to be an effective tool not only in providing quality teaching sessions but also for improved communication with learners and colleagues, sharing learning materials and creating innovative teaching materials to enhance the learner’s understanding. The majority of the faculty had no experience with e-learning previously however were confident about providing education in a virtual environment. This confidence in virtual teaching likely stems from our exposure to personalized learning experiences through online modules and accredited courses on various online platforms [20]. Similar findings were noted in a survey conducted in India with 56% faculty having good knowledge of e-learning with a high degree of acceptability of the benefits of e-learning [21]. These perceptions also reflect the efficiency and user-friendly configurations of commonly used virtual platforms in our institutions such as Zoom, WebEx Microsoft Teams and Google classroom. As noted by Zaiyed et al., with the number of e-learning platforms available it is worthwhile to have an objective comparison to evaluate the most effective platform, as online learning might become an integral part of future medical education [22]. Until such objective evaluations are available, students must be motivated to integrate self -directed learning with easily accessible mentor support in this virtual learning environment to gain understanding and confidence [23].

The limitations of this study include the delivery of the survey to the respondents (which depended on the integrity of servers as well as technical capability). Although the survey was validated with excellent reliability indices, all survey-based studies have inherent limitations due to their subjective interpretation and perceptions. Response rates of post graduate trainees and faculty were low, which we attribute to the increasing workload secondary to the pandemic. This is a cross sectional study which inherently represents a snapshot view and hence it may not be valid over time.

## Conclusion

In conclusion, both trainees and faculty agreed that the COVID-19 pandemic had a significant impact on the provision of medical training in Qatar, with an increased reliance on e-learning modalities. Because of the rapid transition from traditional methods of medical education to e-learning, there was insufficient input from trainees and faculty to effectively develop and guide these changes. Our survey results show that trainees prefer face-to-face education models, and our shift to virtual learning necessitates a redesign of the medical curriculum in terms of delivery and effectiveness in developing a competent medical workforce of the future, while keeping our trainee’s needs and goals in context. As a result, we recommend the involvement of trainees and faculty in the redesign and reframing of these newer modes of delivery of medical education. Our increased focus and use of e-learning will enable us to learn more about the advantages and disadvantages of these activities, allowing us to incorporate and adapt them better (or not use them at all) in the future for medical education. In an era of robotic and remote surgeries, one can't help but wonder what the future of medical education and training holds for tools like virtual and augmented reality and online simulation. In the face of the pandemic, our sudden but forced shift to electronic forms of learning may thus turn out to be a blessing in disguise. However, more research is needed to determine objective outcome differences – such as graduation rates and test scores over time. Students' and faculty's attitudes toward e-learning may also change over time as they adapt to and become more familiar with it and this needs to be looked into in the future.

## Supplementary Information

**Additional file 1: S1Table. **Trainee reasons for preference of models of education. **S2Table. **Faculty reasons for preference of models of education. **S3Table. **Faculty’s concerns about e-learning. **S1Figure.** Scree plot for construct validity. **Appendix A. **Faculty survey Questionnaire. **Appendix B. **Trainee Survey Questionnaire

## Data Availability

The datasets generated and/or analysed during the current study are not publicly available due to information that could compromise the privacy of research participants, but are available from the corresponding author, Dr Merlin Thomas on reasonable request.

## Abbreviations

WCMQ: Weill Cornell Medical College, Qatar
ACGME-I: Accreditation Council for Graduate Medical Education – International
QU CMED: Qatar University—College of medicine
HMC: Hamad Medical Corporation

## References

[1] Murphy B. COVID-19: how the virus is impacting medical schools. Am Med Assoc. 2020. https://www.ama-assn.org/delivering-care/public-health/covid-19-how-virus-impacting-medical-schools Updated MAR 18, 2020. Accessed 3 June 2021.

[2] Desai AN, Patel P (2020). Stopping the spread of COVID-19. JAMA.

[3] Kronbichler A, Kresse D, Yoon S, Lee KH, Effenberger M, Shin JI (2020). Asymptomatic patients as a source of COVID-19 infections: A systematic review and meta-analysis. Int J Infect Dis.

[4] Qatar announces closure of schools, universities over coronavirus. Al Jazeera. https://www.aljazeera.com/news/2020/3/9/qatar-announces-closure-of-schools-universities-over-coronavirus March 9, 2020.

[5] COVID Facilities and Services. https://www.hamad.qa/EN/COVID19/COVID-Facilities-and-Services/Pages/default.aspx. Accessed 12 May 2021.

[6] QU continues educational system through virtual learning. The Peninsula. https://thepeninsulaqatar.com/article/13/03/2020/QU-continues-educational-system-through-virtual-learning. March 13, 2020.

[7] Qatar ministry introduces new blended learning plan for schools. Online Qatar. https://www.onlineqatar.com/news/latest-news/2020/qatar-ministry-introduces-new-blended-learning-plan-for-schools. August 24, 2020.

[8] Singh R, Agarwal TM, Al-Thani H, Al Maslamani Y, El-Menyar A. Validation of a survey questionnaire on organ donation: An Arabic world scenario. Journal of Transplantation, vol.2018, Article ID 9309486,10 pages,2018 10.1155/2018/9309486. 10.1155/2018/9309486 PMC5822804 29593894

[9] Glaser B, Strauss A. The discovery of grounded theory: strategies for qualitative research. EE: UU. Aldine Publishing Company; 1967. 10.1097/00006199-196807000-00014.

[10] Corbin J, Strauss A. 2008. Basics of qualitative research. 3rd edn Thousand Oaks. CA: Sage Publications.

[11] Gishen F, Bennett S, Gill D. BMJ Opinion. Covid-19—the impact on our medical students will be far-reaching.https://blogs.bmj.com/bmj/2020/04/03/covid-19-the-impact-on-our-medical-students-will-be-far-reaching/. Updated April 3, 2020. Accessed 10 May 2020.

[12] Choi B, Jegatheeswaran L, Minocha A, Alhilani M, Nakhoul M, Mutengesa E (2020). The impact of the COVID-19 pandemic on final year medical students in the United Kingdom: a national survey. BMC medical education..

[13] Virtual Teaching Resources. Royal College of Physicians and Surgeons Canada. https://www.royalcollege.ca/rcsite/documents/about/covid-19-virtual-teaching-resources-e. Accessed April 2021.

[14] Reed DS, Hill MD, Justin GA, Giles GB, Santamaria JA, Hobbs SD, Davies BW, Legault GL (2020). Finding Focus in Crisis: Resident-Driven Graduate Medical Education at a Military Training Facility during the COVID-19 Pandemic. Mil Med.

[15] Yadala S, Nalleballe K, Sharma R, Lotia M, Kapoor N, Veerapaneni KD, Kovvuru S, Onteddu S. Resident Education During COVID-19 Pandemic: Effectiveness of Virtual Electroencephalogram Learning. Cureus. 2020 ;12(10). doi:10.7759/cureus.11094. 10.7759/cureus.11094 PMC7581218 33110712

[16] Amir LR, Tanti I, Maharani DA, Wimardhani YS, Julia V, Sulijaya B, Puspitawati R (2020). Student perspective of classroom and distance learning during COVID-19 pandemic in the undergraduate dental study program Universitas Indonesia. BMC Med Educ.

[17] Dost S, Hossain  A, Shehab  M, Abdelwahed  A, Al-Nusair  L (2020). Perceptions of medical students towards online teaching during the COVID-19 pandemic: a national cross-sectional survey of 2721 UK medical students. BMJ open.

[18] Alsoufi A, Alsuyihili  A, Msherghi  A, Elhadi A, Atiyah  H, Ashini A, Ashwieb  A, Ghula  M, Ben Hasan H, Abudabuos  S, Alameen H (2020). Impact of the COVID-19 pandemic on medical education: Medical students’ knowledge, attitudes, and practices regarding electronic learning.. PloS one.

[19] Megan Brooks. COVID -19 Taking a Toll on Med students Survey Shows. Medscape. https://www.medscape.com/viewarticle/939798 Updated Oct 26, 2020. Accessed 10 Nov 2020.

[20] O’Doherty D, Dromey M, Lougheed J, Hannigan A, Last J, McGrath D (2018). Barriers and solutions to online learning in medical education–an integrative review. BMC Med Educ.

[21] Joshi KP, Jamadar D, Dixit R (2020). Perception of faculty toward online teaching and learning in the undergraduate medical students during coronavirus disease-19 pandemic. Int J Med Sci Public Health.

[22] Saiyad S, Virk A, Mahajan R, Singh T (2020). Online Teaching in Medical Training: Establishing Good Online Teaching Practices from Cumulative Experience. Int J Appl Basic Med Res.

[23] PMID: 33088735; PMCID: PMC7534709

[24] Sani I, Hamza Y, Chedid Y, Amalendran J, Hamza N (2020). Understanding the consequence of COVID-19 on undergraduate medical education: Medical students' perspective. Ann Med Surg (Lond).

