# Supplementary material for: A cross sectional survey on the effect of COVID-19 related restrictions on undergraduate and postgraduate medical education in Qatar
Source: BMC Med Educ. 2022 Mar 29;22:212. doi: 10.1186/s12909-022-03268-z (PMC8960666; doi:10.1186/s12909-022-03268-z)
Supplement: Supplementary file 1 — Additional file 1: S1Table. Trainee reasons for preference of models of education. S2Table. Faculty reasons for preference of models of education. S3Table. Faculty’s concerns about e-learning. S1Figure. Scree plot for construct validity. Appendix A. Faculty survey Questionnaire. Appendix B. Trainee Survey Questionnaire [file 12909_2022_3268_MOESM1_ESM.docx]

**Supplementary Files**

**S1 Table: Trainee reasons for preference of models of education**

| Face to Face activity | Combination of all models of education | E- learning only |
| --- | --- | --- |
| Better interaction with mentors and colleagues. Active involvement. | More interactive. | Convenient and easy to catch up on missed educational material. |
| More organised. | Ease of access and convenience if activities are missed due to clinical commitments or illness. | Flexible. One can learn at their own pace. |
| Attention, learning, and retention is more. | Reading the notes and listening to the recordings again gives better understanding of the lectures, while physical presence helps with skill acquisition. | Limits exposure to illness. |
| Direct supervision and discussions. | All senses need to be engaged for effective learning.  Attracts different types of learners. | Eliminate unnecessary traveling time. |
| Acquiring more technical skills. | Better to continue education than not. | Repeated viewing and reinforcement till concepts are understood. |

**S2 Table: Faculty reasons for preference of models of education**

| Face to face | Combination of Modalities |
| --- | --- |
| Maintain focus and demand attention of learners. | Learners have differing learning styles. |
| Engaging, interactive and hence more effective. | Depending on skills / information; different methods of teaching can be combined. |
| Clinical teaching needs to be applied live on patients to acquire knowledge and skills. | Theory can be given online but practical skills need face-to-face sessions. |
| Better evaluation of commitment and effort. | Convenient to provide education during free time. |
| Easy to grasp the mood of the target group with interactive sessions and feedback. | Better utilisation of resources during the pandemic. |

**S3 Table: Faculty’s concerns about e-learning**

| Rank (% respondents from 21 responses) | Reason |
| --- | --- |
| 1 (38) | Requires more formal training and practice to deliver e-learning at the level of face to face activities. |
| 2 (14.2) | The e-learning methods require expertise in technology and may become a hurdle in teaching for older generation. |
| 2 (14.2) | Direct interaction will be difficult. |
| 2 (14.2) | Claiming CPD will not be reliable when learners can leave the camera on and pursue other activities. |
| 2 (14.2) | Difficult to gauge actual engagement. |
| 2 (14.2) | No dedicated space to deliver e-learning and concerns of data confidentiality. |
| 3 (4.8) | Loss of the impact of body language on learning. |
| 3 (4.8) | Technical glitches leading to disrupted learning. |

**S1 Figure:** Scree plot for construct validity


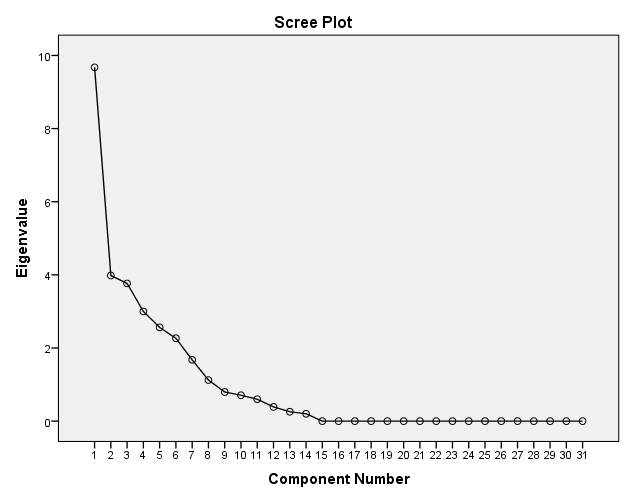

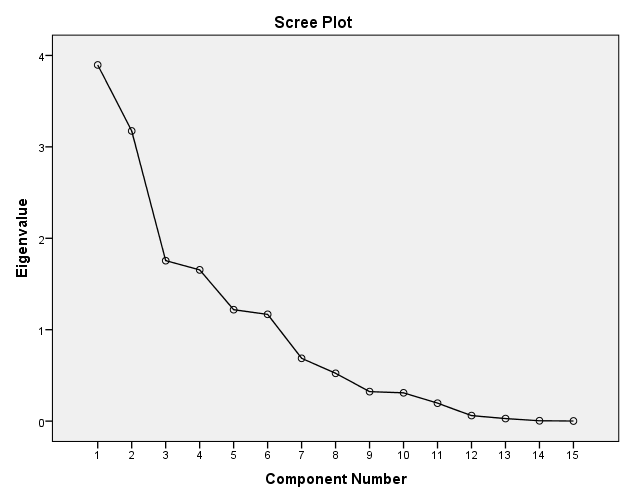

**Appendix A**

**Faculty survey Questionnaire**

# FACULTY SURVEY

1. Which institution do you work at?
2. Qatar University
3. Hamad Medical Corporation
4. Both
5. Your target group of learners include:

Select all that applies

1. Qatar University Medical students
2. Residents
3. Fellows
4. Faculty
5. Others, Specify
6. Since the outbreak of the global pandemic COVID-19 what challenges do you face?

Select all that applies

1. Work – life balance: eg. Increased workload
2. Mental stress
3. Home schooling
4. Relationship issues
5. Self-education (CPD/CME)
6. Others, Specify……
7. Do you think there are barriers to the delivery of education during COVID-19 pandemic?

Yes No

If yes, select your level of agreement with the following statements:

Levels of agreement -Strongly agree /agree /don’t know /disagree/Strongly disagree

- 1. Unavailability of trainees at bedside/classroom
  2. Social distancing measures
  3. Re prioritization of your tasks
  4. Lack of time
  5. Issues around infection control
  6. Others, Specify?

1. Prior to the pandemic what was the frequency of educational activities in your program
2. Daily
3. Weekly
4. Twice a Month
5. Monthly
6. Biannual
7. Annual
8. Since the declaration of COVID-19 based restrictions on March 10^th^, what is the current frequency of your education in comparison to the previous state?
9. Daily
10. Weekly
11. Twice a Month
12. Monthly
13. Biannual
14. Annual
15. Not happening at all
16. In the current COVID 19 pandemic, what is your level of agreement with the following statements?
17. It’s important to focus on providing clinical service than on education.

Strongly agree / agree /don’t know/Disagree /Strongly disagree

1. Continuing medical education is equally important to providing the clinical service.

Strongly agree / agree /don’t know/Disagree /Strongly disagree

1. What models of education were provided by your institution to the trainees before COVID -19 pandemic?

Select all that applies

1. Face to face Didactic lectures: Eg. Morning report, Grand rounds
2. Seminars
3. Workshops
4. PICCO conference/ Journal Clubs
5. Bedside teaching
6. E- Learning

*E-learning, also known as online learning, is obtaining knowledge through electronic technologies and media*

1. Which model of education are you using during the COVID-19 pandemic?

Select all that applies

1. Face to face live lectures/ workshops/ Journal Clubs
2. Bedside teaching
3. Record lectures and broadcast it (asynchronous)
4. Live online lectures (Synchronous)
5. Providing learning materials, such a as links, reference articles etc.
6. Self -directed learning (Encourage trainees to learn on their own)
7. None
8. In general, what type of teaching modality do you find most effective?
   - 1. Face to face (Physical presence of the instructor eg. Grand rounds, morning reports, didactic lectures)
     2. Self-directed Learning (E-resources such as UpToDate and BMJ modules)
     3. Pre-Recorded Archived Lectures
     4. Live broadcasting of lectures (Zoom, Web Ex, Webinar)
     5. Combination of the above

Why do you find this teaching modality most effective? (Optional)

----------------------------------------------------------------------

1. Do you have experience using e-learning as a teaching modality before?

Yes No

1. I am confident in providing education using e-learning software.

Yes No

If No, please specify why?

1. I have not received any technical training on the e -learning software Yes No
2. I am not familiar with the e learning software Yes No
3. I have challenges in understanding and operating technology Yes No
4. Others, Specify
5. To what extend do you think E-Learning can support education?

Levels of agreement -Strongly agree /agree /don’t know /disagree/Strongly disagree

1. Provision of course materials to learners
2. Share course materials with colleagues
3. Present information in front of the class – real time
4. Create innovative teaching materials
5. Develop learners’ understanding of the subject
6. Communicate with learners outside of the classroom
7. Assist in giving one-to-one attention to learners
8. Test learners’ understanding
9. Track learners’ progress
10. Manage individual target setting for learners

**Appendix B**

**Trainee Survey Questionnaire**

MEDICAL TRAINEE SURVEY

1. At which level of Medical Training are you?
   - 1. Medical Student
     2. Internship
     3. Residency
     4. Fellowship
2. What specialty are you training in? (for Residents and Fellows (core program)
   - 1. Internal Medicine
     2. General Surgery
     3. Anesthesia
     4. Obstetrics/ Gynecology
     5. Emergency Medicine
     6. Pediatrics
     7. Radiology
     8. Neurosurgery
     9. Neurology
     10. Cardiology
     11. Orthopedics
     12. Otolaryngology
     13. Ophthalmology
     14. Family Medicine
     15. Geriatrics
     16. Psychiatry
     17. Dermatology
     18. Plastic Surgery
     19. Urology
     20. Pathology
     21. Rheumatology
     22. Pulmonary
     23. Infectious disease
     24. Nephrology
     25. Gastroenterology
     26. Others, please specify
3. How frequently are you visiting your workplace or university during COVID -19 pandemic?
   - 1. Same as before
     2. More than before
     3. Less than before
     4. I do not come at all

If the answer is iii (Less than before) or iv (I do not come at all)

Select all that apply

i) You have a medical condition

ii) You are pregnant

iii) You are out of country and unable to travel back due to the travel restrictions

iv) You have no interaction with patients and can work and learn from home

1. Has the current COVID-19 pandemic negatively impacted your training ?
   - 1. Yes
     2. No

If Yes, How? -------------------------------(Optional)

1. You had to stop attending face to face educational sessions since the COVID -19 Pandemic.
   - 1. Yes
     2. No
2. If yes, what is the reason?

Select all that apply

- - 1. You are serving at the ‘frontline’
    2. Your education program or institution has stopped the activity
    3. Other please specify: ---------------------------------------

1. What was the frequency of educational activities in your program before the pandemic emerged?
   - 1. Daily
     2. Twice weekly
     3. Once weekly
     4. Twice monthly
     5. Monthly
2. Since the declaration of COVID-19 based restrictions on March 10^th^, what is the frequency of your education activities ?
3. Daily
4. Twice weekly
5. Once weekly
6. Twice monthly
7. Monthly
8. Not happening at all
9. What model of education are you receiving during the COVID-19 pandemic?
10. Face to face - lectures/ workshops/ Journal Clubs
11. Bedside teaching
12. Broadcast of recorded lectures (asynchronous)
13. Live online lectures (Synchronous)
14. Provided learning materials, such a as links, reference articles etc.
15. Self -directed learning (Trainers encourage us learn on our own)
16. None
17. Are you currently being trained via e-learning?

Yes No

If yes, to what extend do you agree with the following statements.

Levels of agreement -Strongly agree /agree /don’t know /disagree/Strongly disagree

1. E-learning is enhancing my learning
2. E-learning is enhancing my communication skills (writing skills, verbal communication with colleagues and mentor)
3. E-learning is as effective as traditional learning methods (face to face lectures/seminars/workshops)
4. E-learning helps my learning by integrating many forms of media
5. In general, what type of teaching modality do you find most effective?
   - 1. Face to face (Physical presence of the instructor eg. Grand rounds, morning reports, didactic lectures)
     2. Self-directed Learning (E-resources such as UpToDate and BMJ modules)
     3. Pre-Recorded Archived Lectures
     4. Live broadcasting of lectures (Zoom, Web Ex, Webinar)
     5. Combination of the above

Could you explain why ?...........................................
